# Clinical Audit for Integration of Communicable and Non-Communicable Diseases at the Primary Health Care Level in Tanzania

**DOI:** 10.24248/eahrj.v7i2.737

**Published:** 2023-11-30

**Authors:** Stellah G Mpagama, Nyasatu G Chamba, Kenneth C Byashalira, Albino Kalolo, PendoMartha J Shayo, Kaushik L Ramaiya, Peter Nigwa, Catherine Gitige, Anna Chongolo, Scott K Heysell, Blandina T Mmbaga, Troels Lillebaek, Ib C Bygbjerg, Rachel N Manongi, Dirk L Christensen

**Affiliations:** a Kibong'oto Infectious Disease Hospital, Sanya Juu, Siha, Tanzania, United Republic; b Kilimanjaro Christian Medical University College, Moshi, Tanzania, United Republic; c Kilimanjaro Clinical Research Institute, Moshi, Tanzania, United Republic; d Department of Internal Medicine, Kilimanjaro Christian Medical Centre, Moshi, Tanzania, United Republic; e Department of Public Health, St. Francis University College of Health and Allied Sciences, Ifakara, Tanzania; f Shree Hindu Mandal Hospital, Dar es Salaam, Tanzania, United Republic; g Kilimanjaro Regional Administrative Secretary-Health; h University of Virginia - Division of Infectious Diseases and International Health, Charlottesville, Virginia, U.S.A; i International Reference Laboratory of Mycobacteriology, Statens Serum Institut, Copenhagen, Denmark; j Global Health Section, Department of Public Health, University of Copenhagen, Copenhagen, Denmark

## Abstract

**Introduction:**

Poor quality of health care services remains an important challenge in health care delivery systems. Here, we validate clinical audit tools and describe audit results of selected clinical standards related to communicable disease (CD) and non-communicable disease (NCD) integration at the primary health care level.

**Methodology:**

A multi-methods approach, including a retrospective cohort and cross-sectional design, was deployed concurrently at Health Centres. Separate evaluators assessed the Health Centres using an audit tool and the inter-rater/inter-observer reliability was estimated. The extent of adherence to clinical standards was measured in proportions for: infection prevention control, tuberculosis (TB) diagnosis including advanced TB/Human Immunodeficiency Virus (HIV), the diagnosis of chronic lung diseases, and the bidirectional screening and clinical management of TB and Diabetes Mellitus (DM).

**Results:**

The inter-rater reliability for the clinical audit tools based on 130 individuals' charts was 99.5% (CI:99-100). The total estimated maximum score for infection prevention control was 114 and on average health centres scored 42 (37%). Only 3 (4%) of 80 individuals' medical charts with unexplained productive cough were evaluated for TB. None of the 24 individuals with HIV infection medical charts had vitals measured and only 6 (25%) patients with advanced HIV had a TB test performed, whereas 4 (17%) had a cryptococcal antigen test, and 1 (4%) had a chest radiograph. Also, 24 patients' chart from documented HIV negative with chronic cough had no records of spirometry or peak flowmeter or a chest radiograph. However, a diagnosis of asthma and chronic obstructive pulmonary disease as made in 17 (71%) and 7 (29%), respectively. TB was confirmed for 102 patients among whom only 12(12%) were screened for DM. The DM clinics had no TB presumptive registers. Patients with TB/DM (n=2) had a glycated haemoglobin (HbA1c) measurement done and received appropriate management.

**Conclusion and recommendation:**

The developed clinical audit tools were reliable and could contribute to quality measurement for metrics-related integration of CD and NCD in Tanzania. Further investigations will determine if the clinical audit tools widely used in cycles can improve the quality of care in health care delivery systems.

## BACKGROUND

Although most of the East African countries have documented control measures to the increasing convergence of non-communicable diseases (NCDs) such as diabetes mellitus (DM) and chronic lung disease, and communicable diseases (CDs) such as human immunodeficiency virus (HIV) and tuberculosis (TB), it remains a considerable challenge to implement the recommended measures within primary health care settings and current health care delivery systems. ^1^ The East African region consists of low- and middle-income countries (LMICs) with limited budgets for alteration of existing national health systems. It is recognized that countries in the region contribute 8 million excess deaths every year attributed to poorly functioning health services.^2^ Poor quality of care not only leads to adverse outcomes such as increased death toll, unnecessary suffering, persistent symptoms, and loss of function, but individuals seeking care in such systems may permanently lose trust and confidence in traditional biomedical care as delivered.^3^ Various initiatives to mitigate poor quality of health services in the East African region have already been described. For instance, in Tanzania a new supportive supervision approach has been created and has shown promising results at the primary health care level.^4^

Recognizing the challenges of health care delivery systems particularly at the intersection of CDs and NCDs in Tanzania, we designed adaptive diseases control expert programme (ADEPT), a model with two important drivers to support integration of health care services. ^5^ The first domain was a stepwise training approach for knowledge and skills improvement of the front-line health care providers, which included innovations such as e-learning, whereas the second domain was a continuous learning approach that included implementation research and development of clinical audit.^5^ In general, a clinical audit consists of measuring a clinical outcome or process against a well-defined evidence based standard set in order to identify changes required to improve the quality of care. The clinical audit has already shown that it may drive improvement in the quality of care in LMICs, and therefore may be useful to develop and evaluate in the Tanzanian setting.^6^ Our objective in this study was to validate the developed clinical audit tools, and describe the audit results of selected clinical standards related to CD and NCD integration at the primary health care level.

## METHODS

### Study design and settings

This was a multi-methods study including a retrospective cohort reviewing participants' medical records from 1 January to 31 December of 2020, and a cross-sectional design to assess infection prevention during one survey period. The clinical sites were Majengo and Pasua Health Centres located in Moshi, Kilimanjaro.

### Description of the steps of conducting audit using the Clinical Audit Tools

#### Step 1. Preparations for the audit

We selected the topics for clinical audit at the primary health care level in Kilimanjaro region. The facilities operate under the regional administration and local government authority. We designed tools against the available clinical standards. ^7–9^ The topic and the rationale for including the topic is described in Table 1.

**TABLE 1:** Selected Clinical Audit Topics and the Rationale for Audit in Relationship with Intersection of Communicable and Non-communicable Diseases

Clinical Audit topics	Rationale
1 Infection Prevention Control (IPC) at the health facility settings	PHC are organizing NCD care, thus effective implementation of IPC will prevent nosocomial transmission of infectious diseases to individuals with NCD [16]
2 Screening for TB at the general outpatient settings	1/3 – 2/3 of individuals presumed with TB attend health care facilities but are not evaluated accordingly [20]
3 Screening for TB in people living with advanced HIV	45% of individuals with advanced HIV dies from TB diagnosed postmortem [21]
4 Screening for non-communicable chronic lung diseases in individuals evaluated for pulmonary TB and negative	48.5% (39-58) individuals presenting with chronic respiratory symptoms and assessed for pulmonary TB are bacteriological negative and without other signs of PTB like e.g., chest found radiograph [24]
5 Bidirectional screening for TB in people affected with DM	There is a high co-prevalence of TB and DM in low- and middleincome countries. DM prevalence in TB ranges 10–30%, whereas the prevalence of TB in DM ranges 0.1 – 6% [25]
6 Clinical management of dual TB Er DM and other associated comorbidities	DM is associated with increased risk of poor TB treatment outcomes, particularly mortality and increased risk of developing multidrug resistant TB [26]

Definition of terms

IPC-Infection Prevention Control: PHC – Primary Health Care: NCD – Non communicable Diseases: TB – Tuberculosis:

DM-Diabetes Mellitus

#### Step 2. Selection of the clinical standards and indicators

The clinical standards were selected from clinical guidelines reflecting the most current recommended management in the country. Three clinical guidelines steered the audit tools: Infection prevention control, ^7^ management of TB in Tanzania including the intersections of DM and HIV, ^8^ and the management of NCDs at the primary health centre level, specifically non-communicable chronic lung diseases (asthma, chronic obstructive pulmonary diseases and post-TB lung disease). ^9^ Criteria from sites implementing the ADEPT project and the ultimate measurement of sites meeting these criteria were reported in percentages. The criteria described as clinical standards are described as follows;

- Health facility has a “transmission-based precautions”-system for patients documented or presumed to be infected with highly transmissible or epidemiologically important diseases.
- All persons with otherwise unexplained productive cough should be evaluated for TB. The most common symptom of pulmonary TB is persistent productive cough, often accompanied by constitutional symptoms, such as fever, night sweats, coughing up blood (haemoptysis), chest pain, shortness of breath and weight loss.
- People living with HIV (PLWH) presenting with signs and symptoms of sepsis need to be evaluated and managed for TB or sepsis or both.
- Any individual evaluated at the health facility and found to be bacteriologically negative (smear or Xpert MTB/RIF) and clinically non-PTB needs to be further evaluated for non-communicable chronic lung diseases (Asthma, chronic obstructive pulmonary disease (COPD), and post TB lung disease after excluding COVID-19.
- All new TB patients should be screened for diabetes at the start of TB treatment using the DM screening questionnaire to identify those with symptoms and signs of DM. Random blood glucose (RBG) and fasting blood glucose (FBG) tests should be performed per the algorithm for diagnosis of diabetes among TB patients.
- Management of patient with dual TB and DM either co-infected with HIV or not, the treatment will follow the standards TB treatment guideline and DM management will be in accordance with HbA1c levels as follows:

The lower limit of the acceptability target for meeting criteria was 80%.^10^

#### Step 3. Data collection

The variables and score for each criterion was set (Annex 1). Patients' file numbers were included in the database for easy traceability but accessible only to approved clinical staff, whereas other information identifying the participant were not included for protecting patients' privacy.

#### Step 4. Comparison of collected auditable data

Each tool was collected by two people on separate days at intervals of one week. This process was undertaken to determine if tools were reliable and measurements were estimated correctly with different individuals at different time intervals.

### Sample Size and Sampling Strategy

Estimates were given for an alpha of 0.05 and a beta of 0.20. For an inter-rater reliability, at least 40 medical charts per health centre were sufficient to estimate a minimum acceptable level of reliability as described elsewhere.^11^ Depending on the clinical audit criterion, we audited 9 and 7 items for the infection prevention control at the out- and in-patients setting, respectively. The items included availability of infection prevention guideline, hand hygiene standard operating procedure, triage practices, clinic organization, and sitting arrangement and ventilation. Clinical audit of records particularly for the clinical conditions included upper and lower respiratory tract infections (pneumonia), vital signs including temperature, respiratory rate, pulse rate, and clinical features such as inability to walk unaided, CD4 count <200 cells/µl in individuals with advanced HIV, and non-communicable chronic lung disease at the clinics or inpatients. A random selection of at least one patient's file per month with one of the clinical features and all clinical documents of patients with TB and DM were examined to assess implementation of the bidirectional screening for TB and DM. For all participants with dual TB and DM, medical records were collected for clinical audit. The selected health centres 2 (7%) were part of the Kilimanjaro – Moshi participating ADEPT health facilities, and were purposively selected to affiliate with Kibong'oto Infectious Diseases Hospital, which was leading the assessment.

### Clinical Audit Data Sources

Data for clinical audit were accessed in the District Health Information Software (DHIS)-2, HIV registry, TB registry, and other informal documents used to document patients' management at the clinics. ^12^ The clinical audit questionnaires were written and information filled out in English.

### Data Entry and Validity Check

An electronic questionnaire with measures was created in Microsoft Excel. Validity check was set to avoid wrong numbers by using a dropdown menu. Trained auditors entered data directly in the electronic questionnaire. Data were analyzed using Microsoft Excel and Statistical Package for Social Science.

### Statistical Analysis

We calculated the inter-rater and inter-observer reliability and agreement percentage of the completed data and Cohen's κ value. A κ value between 0.00 – 0.20 was classified as ‘slight’, between 0.21–0.40 as ‘fair’; between 0.41 and 0.60 as ‘moderate’ between 0.61–0.80 as ‘substantial’; and between 0.81 and 1.00 as ‘almost perfect’. We used percentage and descriptive methods for the remaining calculations.

### Ethics

This study had ethical approval from the local research ethics committee with reference KNCHREC003 and the National Health Research Ethical Committee (NIMR/HQ/R.8a/Vol.IX/2988). This manuscript is reported according to the standards for reporting reliability and agreement studies.^13^

## RESULTS

### Characteristics of the Health Centers

The prescribing officers (medical, assistant medical, clinical, and assistant clinical) was 11 and 13, whereas the number of nursing officers (nurse, assistant nurse) was 16 and 18. The number of laboratory staff was 4 and 5 and pharmacy staff was 1 and 2, respectively for Majengo and Pasua. The mean number of individuals attending the health centres for any reason were 23,123 ± 325 over the course of the 2020 calendar year. Among the top 10 diseases reported for which patients sought care, upper respiratory tract infection was the leading disease, while pneumonia was between the third and fourth position in all health centres. The mean number of individuals with upper and lower respiratory tract infection was 11,782 ± 520 and 428 ± 46, respectively.

### Data Completeness

Of the 80 individuals documented to present with cough, none (0%) of the patients' charts were recorded with additional TB presumptive as shown on Figure 1a. Furthermore, for 24 individuals with cough and evaluated for TB and found to have negative TB test results, none of the medical charts showed clinical features for noncommunicable chronic lung disease diagnostic algorithm Figure 1b. Also, among 96 examined individual charts at the HIV clinic, none was examined for the danger signs, reducing the likelihood of implementing the TB diagnosis algorithm in advanced HIV as portrayed in Figure 1c. Likewise, of 12 individuals with CD4c count < 200 cells/µl, none had documented measurements of vital signs and only 2 (17%) had documentation of cough and fever and other conventional TB symptoms.

**FIGURE 1a:**
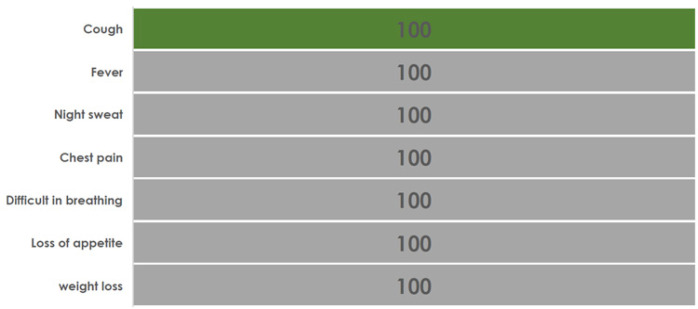
Incomplete Documentation of Clinical Features in the Records for Individuals Presumed Pulmonary TB Attending the Outpatients Clinic

**FIGURE 1b:**
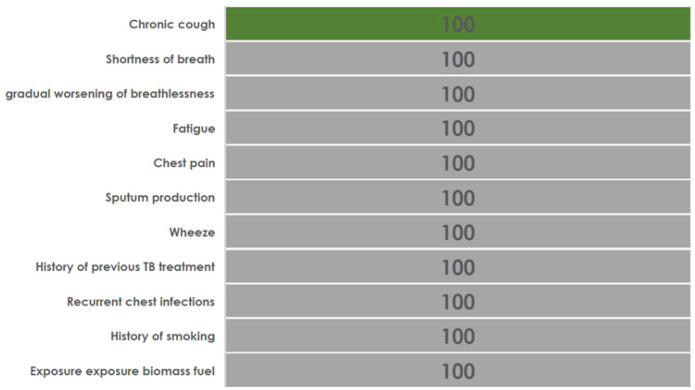
Incomplete Documentation of Clinical Features in The Records for Individuals with Presumed Chronic Lung Disease Attending the Outpatient Clinic

### Inter-Rater Reliability and Adherence of Health Facilities to the Clinical Standards

Of the 130 individuals' clinical records from all clinics, the inter-rater reliability for the clinical audit tools was 99.5% (CI:99-100) almost perfect. There was no difference in reliability between clinics (*p>0.99*).

The total expected score for the standard “Health facility has a transmission-based precautions system for patients documented or presumed to be infected with highly transmissible or epidemiologically important diseases” was 114 and health centers scored on average a 42/114 (37%). Areas that had limited score in all facilities included infection prevention control (IPC) guideline and standard operational procedure for sputum collection as shown on Table 2.

**TABLE 2:** Infection Prevention Control Clinical Standard

Parameter assessed	Expected score	Score (%)
Availability of the Infection Prevention Guideline	18^#^	0(0%)
Availability of sputum collection SOP	18^#^	0(0%)
Hand hygiene SOP	18^#^	7(39%)
Hand hygiene practices	12^η^	8(67%)
Triaging of coughers (outpatients only)	4^§^	1(25%)
Clinics organization (outpatients only)	6^¥^	4(67%)
Adequate ventilation	12^$^	9(75%)
Sitting arrangement	8^&^	6(75%)
Health education on TB-IPC	18^@^	7(39%)
Total score	114	42(37%)

# The total score 4 clinics (2-TB, 2-DM and 2-inpatient settings in all facilities) each with a maximum score of 3 meaning having a IPC or SOP document that is accessible in with evidence of training of health care providers

η Adequate hand washing facilities with running water and soap/sanitizer

§ Only DM clinic was assessed for two health facilities

¥ Clinic organized with a senior or specialized patient to attend individuals with multimorbidity particularly TB/DM

$ Adequate ventilation with cross ventilations

& Sitting arrangement between patient and clinician and should be cross ventilation

@ Health education provided and documented

Definition of terms

SOP – Standard Operating Procedures: IPC-Infection Prevention Control

Eighty out of 130 individuals presented with cough and were examined for the clinical standard 2 stating “All persons with otherwise unexplained productive cough should be evaluated for tuberculosis”. Only 3(4%) of these had TB evaluated by additional recommended diagnostics of smear microscopy or the rapid molecular test of XpertMTB/RIF.

Likewise, another clinical standard stating “People living with HIV presenting with advanced disease with signs and symptoms of sepsis need to be evaluated and managed for TB or Sepsis or both” was assessed in 24 selected patients. Only 6(25%) of these patients had a TB test performed (smear microscopy or XpertMTB/RIF), 4(17%) had a Cryptococcal antigen test, and 1(4%) had a chest radiograph.

Twenty-four selected patients with HIV negative test results were examined for the clinical standard stating “Any individual evaluated at the health facility and found to be bacteriologically negative (smear or Xpert MTB/RIF) and were not diagnosed with TB with other diagnostics such as chest radiograph need to be further evaluated for Non-Communicable Chronic Lung Diseases (Asthma, Chronic Obstructive Pulmonary Disease (COPD), and Post TB Lung Disease after excluding COVID-19.” None of the patients had a spirometry or peak flowmeter or a chest radiograph performed.

However, a diagnosis of asthma and COPD was made in 17(71%) and 7(29%) respectively. Patients received bronchodilators 12(50%), antibiotics 10(42%) or inhaled steroids (8%).

Likewise, a clinical standard “A new TB patients should be screened for diabetes at the start of TB treatment.

RBG and FBG tests should be performed per the algorithm for diagnosis of DM among TB patients” was evaluated in 102 out of 102 confirmed TB patients. One patient had known DM and thus excluded, and of the remaining 12(12%) were screened for DM. None of the patients had body mass index estimation as height was only measured in 1% although weight was recorded in 75%. Similarly, screening for TB in DM clinic was examined with a standard “All diabetes patients should be screened for TB at the time of diagnosis and at follow-up visits”. The DM clinics had no TB presumptive registers and was not possible to measure the practice.

A total of two identified patients with TB/DM were found in one of the health facilities. The examined standard was “Management of patient with dual TB & DM either co-infected with HIV or not, the treatment will follow the standards TB treatment guideline and DM management will be in accordance with HbA1c levels as follows.” Patients had measurements of HbA1c level, and received appropriate management according to the level of HbA1c. One patient was assessed for complications although only partially which included full blood count, renal and liver functions. Complications that were not assessed included diabetic foot, neuropathy and retinopathy.

## DISCUSSION

Findings show that the developed clinical audit tools had excellent inter-rater reliability for the health facilities studied, and that they identified numerous opportunities for quality improvement in the health service delivery and integration of CD and NCD management beyond the first phase of initial knowledge dissemination to front-line health care workers. While the suboptimal performance of health centres in meeting clinical standards implementation was not new in the Tanzanian setting, the extent to which facilities were underperforming was unknown.^14,15^ This current study provides a tool for increasing accountability in quality improvement, and the results are immediately deliverable to the health centres themselves and other relevant stakeholders.

Notably, the clinical audit tool found that the organization of health centres partially adhered to the IPC standards for visitors and patients to reduce nosocomial infections. This finding follows a large nationwide report with more than 2,000 health facilities in Tanzania, which also showed that partial adherence to IPC standards was widespread and that inadequate compliance of health care workers to hand hygiene and disinfection, particularly at the outpatient settings, was very common.^16,17^ However, the clinical audit tool in the current study allows facility level data to be delivered to those in the front-lines to better determine root causes, and whether quality of care can be improved with alteration in practice (unwillingness improved with behavior change incentives) or improved with resource allocation (facilities remodel or assuring adequate stock of disinfectant or supplies for hand hygiene).^18^

Currently, it is becoming increasingly clear that advances in technologies and medical sciences, particularly for TB, HIV and those infections in the presence of NCD, has led to an equally momentous change in the recommendations on the clinical management of patients, so that front-line health systems have been unable to match in implementation. ^19^ The clinical audit tool has served to improve service delivery system performance in other similar settings, particularly when patients and healthcare workers have retained a participatory role. For instance, in Ethiopia, the clinical audit in TB clinics showed approximately 50% of TB patients received substandard clinical management, but this included both over and under prescribing of anti-TB medications highlighting areas for correction to limit unnecessary treatment and toxicities, and improve treatment failure for actual TB disease, respectively.^20^ Likewise, a multi-country clinical audit for TB conducted in Latin America showed a considerable improvement in the quality of clinical care with subsequent use of the audit tool, particularly for resource limited settings.^6^ The authors highlighted key factors that influenced success included participatory leadership and TB programme support, whereas barriers included stigma and frequent changes of healthcare personnel and policies.^6^

One of the more surprising findings of this clinical audit was that for a considerable proportion of individuals presenting with cough and presumed pulmonary TB, additional symptoms of fever, night sweat, chest pain, difficulty in breathing, loss of appetite and weight loss were not documented, even when additional presence of those symptoms may incline one towards TB treatment in the absence of confirmed TB diagnostic testing, but at a minimum should prompt diagnostic testing. However, these individuals rarely had a test performed for pulmonary TB. Our findings were even more striking in under-performance than a previous report from Tanzania that showed 42% of individuals with clinical features suggestive of pulmonary TB who had sought care in health facilities with TB diagnostic capacity, were not evaluated for TB. ^21^ Regular audit cycles may identify correctable local factors driving these observations, but we also acknowledge that the audit may have identified the poor utility of the initial phase of knowledge and skills building within the ADEPT model.

Updates on the clinical features including tachycardia, tachypnoea, hyperthermia, inability to walk unaided, or CD4 count below 100, were included for presuming TB particularly in individuals with advanced HIV. It is expected that HIV clinics include a practice for measuring vital signs that would prioritize screening of TB in that high-risk group. Unfortunately, health centres did not measure the vital signs. Whether this is merely negligence or lack of thermometers was not further evaluated. Yet, this signifies the need of the accountability system that will reinforce the best practice that would otherwise reverse the current death rate of 40% of HIV-related facility-based deaths in adults. ^22^

Our findings also highlight how historically TB and TB/HIV services have been operationalized in a vertical approach. Currently, there is a need of integrating TB or TB/HIV with other NCDs such as chronic lung diseases and DM. ^23^ A previous study in Tanzania showed low readiness of health facilities in integrating TB and chronic lung diseases particularly on limited training of health care workers, lack of guidelines for chronic lung disease management and lack of equipment such as spirometry and peak flowmeters for supporting diagnosis. ^15^

Globally, there is a call for bidirectional screening of TB and DM. ^24^ This clinical audit revealed that TB patients were rarely screened for DM. Despite availability of programmatic presumptive TB registry for nationwide use, this registry was not available in DM clinics, and therefore the practice not documented. One health centre in the present study had HbA1c equipment and the two individuals identified with dual TB/DM were monitored by HbA1c. The shortcoming of this study is that it is largely descriptive and cannot provide the causal effect relationship. Yet, it relies on what is available rather than collecting the most valid data. Regardless the clinical audit, if deployed is an accountability tool to improve the quality of care in the clinics.

In conclusion, we have developed reliable clinical audit tools that can assist in quality measurement for metrics related to CD and NCD integration in Tanzania. Further studies will show, that if these clinical audit tools are widely used on a regular basis with healthcare worker and patient participation, then improvement in quality of care will follow.
